# Primary Mucosal Melanoma of the Stomach

**DOI:** 10.1155/2018/6040693

**Published:** 2018-08-12

**Authors:** O. Phillips, A. Higdon, R. Colaco, H. Fish

**Affiliations:** ^1^St. George's University School of Medicine, West Indies, Grenada; ^2^Department of Surgery, Trinitas Regional Medical Center, Elizabeth, NJ, USA; ^3^Division of Surgical Pathology, Trinitas Regional Medical Center, Elizabeth, NJ, USA

## Abstract

Considered to be rare, mucosal melanomas are rare type of melanoma that are found on mucosal surfaces and are primary or metastatic in origin. We report a case of a 66-year-old Hispanic female who presented with vague abdominal pain and upon further endoscopic work-up revealed 2 gastric lesions. Endoscopic biopsy results revealed gastric melanoma in the distal lesion. A PET/CT scan indicated it to be suspicious for the primary site of metastasis but was ultimately diagnosed as a benign nevus on biopsy. An extensive clinical exam showed no other probable sites of origin. The patient underwent a subtotal Billroth II gastrectomy and enterostomy tube placement for temporary feeding. Primary melanoma of the stomach is an exceptionally rare occurrence with limited cases that can be accounted for in literature; thus we report this case for review.

## 1. Introduction

Melanoma is the 6th most common cancer in North America [1]. The incidence of cutaneous melanomas continues to increases in the United States and is correlated with exposure to ultraviolet radiation in non-Hispanic whites [2]. However, exposure to ultraviolet light is not seen as a risk factor in mucosal melanomas since the mucosa is not exposed to UV light. No other risk factor for mucosal melanomas has been clearly identified except for those originating from the oral cavity that has been associated with cigarette smoking [3, 4]. Even though melanocytes are predominately cutaneous, they can be found in various other locations such as the oral pharynx, vulvovaginal, and anorectum [3].

As stated, mucosal melanomas are found to be primary or secondary in nature. Primary mucosal melanomas are usually seen in older populations with an average age of 70 years and accounts for one percent of all melanomas [5–7]. Primary sites of mucosal melanomas can also occur along the gastrointestinal tract, however; they are more commonly in the oropharynx [32.8%], anal canal [31.4%], and rectum [22.2%] [3, 8]. They are more rarely found in esophagus [5.9%], stomach [2.7%], and small bowel [2.3%] [3, 8]. Most lesions found along the gastrointestinal tract are metastatic, with the most frequent locations of metastasis being the small intestine, followed by the colon and stomach at the time of presentation [9, 10]. It is rare to find primary gastric melanomas and specific diagnostic criteria are needed in order to be conclusive [11]. The pathogenesis and etiology of mucosal melanomas have not been clearly identified as melanocytes are not part of the innate cellular constitution of the stomach epithelium [12]. To date, the knowledge of any contributing risk factors continues to be elusive [8]. In general mucosal melanomas are considered to have a poor prognosis with a five-year survival of twenty-five percent [5].

## 2. Case Report

A 66-year-old Hispanic female presented with vague abdominal pain and exertional chest pain. She had a ten-year history of worsening epigastric pain attributed to gastritis that was treated with Dexilant. A general physical exam was done and did not reveal any significant abnormalities; she also denied any fevers, chills, weight loss/gain, or change in bowel habits. Environmental history was noncontributory as she denied extensive sun-exposure or use of tanning beds and reported that she regularly would use sunscreen with a 30 SPF. Still, there was an extensive past medical history for anemia, diabetes mellitus type II, coronary artery disease, asthma, hypertension, hyperlipidemia, hemorrhoids, and osteoarthritis. She also had a broad surgical history, involving a coronary artery stent in the left anterior descending artery [2016], percutaneous transluminal coronary angioplasty [2016], tubal ligation [1985], cholecystectomy [2007], bladder prolapse repair [2012], rectal prolapse repair [2012], hysterectomy [2012], and total right knee replacement [2013].

There is no report of primary tobacco use; however, she does report an extensive second-hand smoke exposure as a result of her biological father. Her family history is significant for multiple cancers, with her father dying of cancer related complications from a head and neck malignancy at the age of 65. The patient's sister died of breast cancer at the age of 44 and half paternal sister also died of breast cancer at the age of 38. Lastly, her brother died of colon cancer at the age of 70 and her mother's death at the age of 67 was due to a myocardial infarction unrelated to cancer. Interestingly, her eldest daughter was diagnosed with melanoma found in a cutaneous lesion, in 2016. Her physical exam did not reveal any concerning skin lesions or palpable abdominal lesions and her vitals were stable.

**Figure 1:**
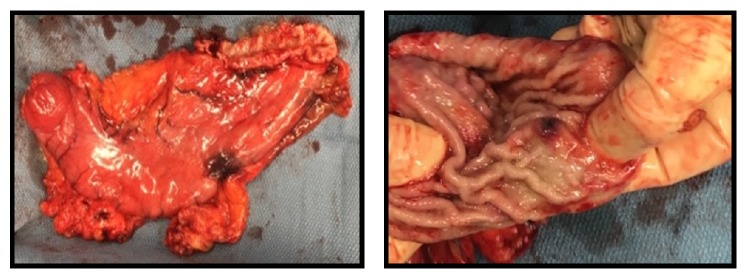
Left: gross whole specimen of the subtotal gastrectomy specimen, 14 cm in length. Preoperatively marked on endoscopy with ink along the greater curvature. Right: proximal lesion near the serosa measured to be 1.1cm x 1 cm was found to be normal gastric mucosa. Distal and proximal margins of the resection were found to be negative.

**Figure 2:**
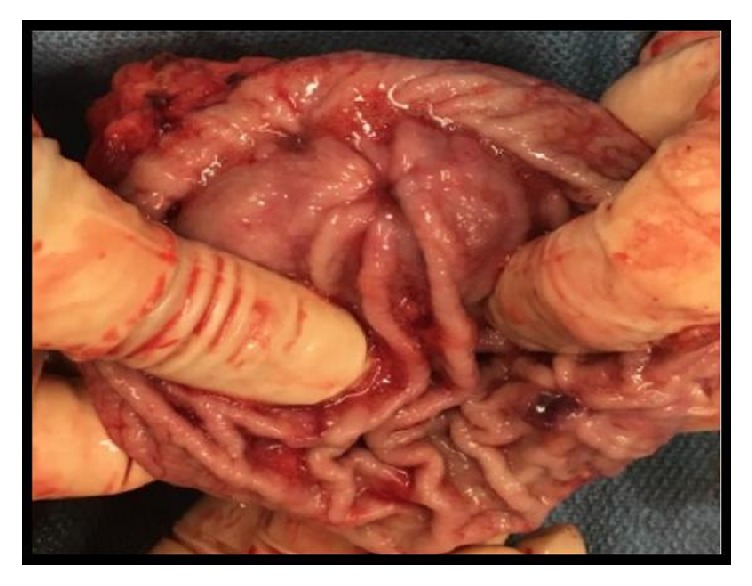
Subtotal gastrectomy distal lesion measuring 0.7 cm x 0.7 cm x 0.5 cm. The lesion was found to be malignant melanoma of the stomach that is invasive into the submucosa. No lymphovascular invasion was identified. Six negative lymph nodes appreciated with the specimen.

A near total gastrectomy was performed and multiple adhesions from prior surgeries were divided at the beginning of the procedure. A thorough surgical exploration of the abdomen demonstrated no lesions of the liver. The gastric lesions, having been preoperatively marked on endoscopy, were inspected. The proximal lesion was near the GE junction and another was located more distally closer to the antrum.  Figure 1 demonstrates the proximal lesion that was located near the greater curvature of the stomach.  Figure 2 displays the distal lesion marked preoperatively located near the antrum of the stomach with no surrounding necrosis or bleeding but was significant for raised mucosal margins in a wheel pattern.

**Figure 3:**
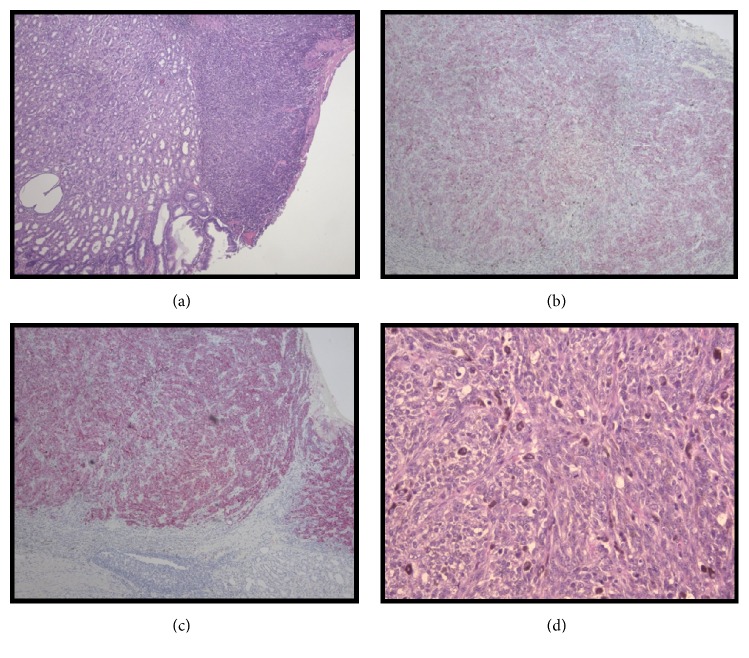
*Histological Specimens of Gastric Melanoma.* Histological sections of gastrectomy specimen demonstrating primary gastric melanoma. (a) Right side of the image depicts neoplastic cells that do not invade past the submucosa that still retains normal gastric architecture (H&E x100). (b) Primary gastric melanoma positive stain for Melanin A (H&E x100). (c) Primary gastric melanoma positive stain for HMB-45 (H&E x100). (d) Malignant melanocytic neoplasm producing brown melanin pigment with a cherry red nucleoli and mitotic figures (H&E x 400).

**Figure 4:**
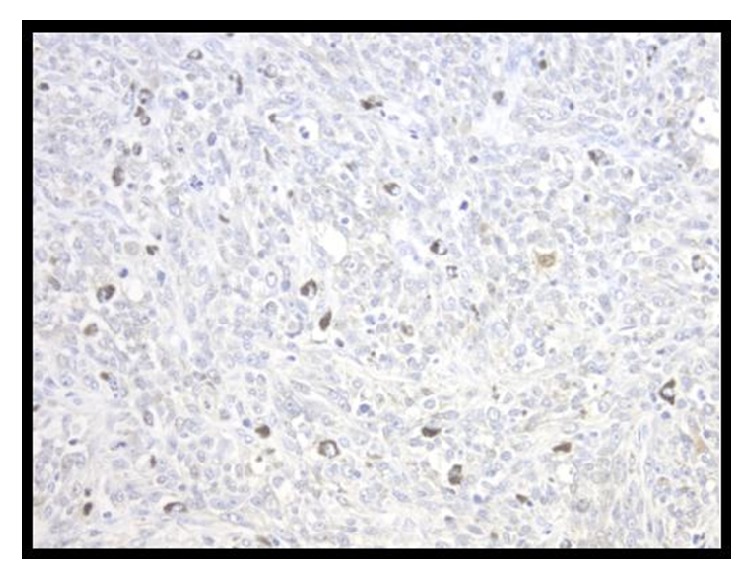
*Histological Specimens of Gastric Melanoma.* Primary gastric melanoma positive stain for S100. Weak and high intensity staining are seen throughout the section (H&E x100).

Reconstruction was performed with a Billroth II anastomosis. Due to the almost complete removal of the stomach, a pouch was created via a jejunojejunostomy. A temporary feeding tube was placed distal to the anastomosis. No blood was transfused through this procedure and the estimated blood loss was 200 ml. The pathology associated with the gross specimens is seen in Figures 3 and 4. They are associated with the second lesion as the first was found to have normal gastric mucosa. The lesions were positive for three significant immunological markers for melanoma, Melanin A, HMB-45, and S100 [13]. Though S100 has been found to have higher sensitivity but much lower specificity for melanoma [13].

Her immediate postoperative course was unremarkable except for a fever of 101 F during the first 24 hours after surgery, which returned to 98.7°F about 9 hours after the initial fever spike in temperature. She remained afebrile for the remainder of her hospital stay. Overall, the patient progressed as expected postoperatively and tolerated her temporary tube feedings well. An upper GI series on postoperative day 5 showed no anastomosis leakage; thus a postgastrectomy diet was started soon thereafter. After a continued unremarkable postoperative course she was discharged on postoperative day 7 to a subacute rehab.

## 3. Discussion

Mucosal melanomas are a very rare type of melanoma and their etiology and pathogenesis continue to remain elusive. Current literature does not have specific staging for primary mucosal melanomas, though the American Joint Committee on Cancer consider mucosal melanomas of any origin are applicable to the current guidelines used for the following mucosal melanomas: head, neck, and vulva (11). This classification system is depicted in Table 1(11).

Lagoudianakis et al. established specific diagnostic criteria for primary malignant melanoma of the gastrointestinal tract [11]. The criteria consist of a lack of any primary sites of melanoma; the patient should have no history or concurrent skin or any other organ removal of a melanocytic lesion, a lack of any adjacent organ involvement, and finally disease free survival for at least 12 months after surgical resection [11]. The last criterion is of critical importance in order to clearly distinguish between primary and metastatic lesions since “50% of patients with stage IV melanoma or visceral disease from unknown primary will have died at 12 months from diagnosis” [11]. This further confirms our diagnosis since at this point in time the patient is currently thirteen months postop.

Additionally, our patient's lack of physical exam findings with a positive histopathology for S100, Melanin A, and HMB-45 suggests that this patient has a true primary gastric melanoma. Overall, even without clear indication of metastasis, the treatment of choice for mucosal melanomas is surgical resection with clear margins [3, 14]. Current literature states that patients with primary gastric melanomas might benefit from additional adjuvant therapy after the initial surgical resection if anatomically possible [5]. It is imperative that we investigate genetic factors in order to further advance chemotherapies combined with surgical resection in order to give patients the best chance for survival. The poor prognosis that is seen with mucosal melanomas is mostly associated due to its late presentation. However, in patients that present early, without invasion through the basement membrane, as in our patient, or possibly even patients who present late, combined chemotherapy with surgical resection could extend survival.

Moreover, the patient's family history of head and neck, breast, and colon cancer as well as cutaneous melanoma implores us to explore possible genetic elements that may have played a role in the development of her disease. For example, CDKN2A is a cyclin dependent kinase inhibitor and encodes for specific tumor suppressor proteins [15–17]. Early onset cutaneous melanomas have been found to have a familial component involving the CDKN2A gene which also has strong associations with “hereditary nonpolyposis colon cancer, familial adenomatous colon cancer, and familial breast cancer” as well as both cutaneous melanoma and mucosal melanomas [16, 18, 19]. Interestingly, mucosal melanomas show even higher rates of CDKN2A mutations than cutaneous melanomas [3, 15–17]. This suggests that loss of function of the CDKN2A gene has an even higher genetic correlation with mucosal melanoma than cutaneous melanoma. Research has indicated that CDKN2A mutations along with KIT oncogene mutations that are seen in about 39% of mucosal melanomas are more common than other mutations that have a strong association with melanomas such as BRAF [3, 16, 20]. BRAF is one of the most significant gene mutations in cutaneous melanoma but is rare in mucosal melanomas and is only seen in about 10% of patients [3]. Though BRAF inhibition has been shown to be advantageous in patients with cutaneous melanoma its potential as an adjuvant therapy in mucosal melanomas patients that do carry the mutation has not been investigated [3]. It has been suggested that the tumor suppressor gene CDKN2A and KIT protooncogene could be key regulators in the pathogenesis of mucosal melanomas [3, 21]. That being said the CDKN2A and KIT gene would be excellent target for further research as potential adjunct dual-targeted chemotherapy for mucosal melanomas.

## 4. Conclusions

Given the fact that there are very few cases regarding primary mucosal melanomas of gastric origin reported in literature, primary gastric melanomas can be considered to be rare malignancies that can present in a number of different ways [8]. Most often it has many nonspecific characteristics in its presentation. According to Anupama Ravi the most common presenting symptoms involve upper gastrointestinal bleeding, anemia, and weight loss [12]. Due to high occurrence of metastatic melanoma to the GI tract, it is paramount that a thorough physical examination is performed as well as other diagnostic criteria before the diagnosis of primary gastric melanoma is given. In the end, early detection of primary gastric melanoma and surgical resection are the initial approaches to a patient who is suspected of having a primary gastric melanoma.

## 5. Limitations

The limitations to this study are mostly concerned with the proper physical examination to confirm the lack of primary lesion. The patient was thoroughly evaluated to confirm the lack of any skin lesions that could have been a primary site of metastasis and none were found. However, the patient had not been evaluated by an ophthalmologist. Therefore, an ocular melanoma at this time has not been ruled out.

## Figures and Tables

**Table 1:** Basic Classification System of Mucosal Melanoma. AJCC_TNM Classification for Mucosal Melanoma.

**Primary Tumor**	
T3	Mucosal Disease

T4a	Moderately advanced disease. Tumor involving deep soft tissue

T4b	Very advanced disease

**Regional Lymph Node Involvement**	

NX	Regional Lymph nodes cannot be assessed

N0	No regional lymph node metastasis

N1	Regional lymph node metastases present

**Distant Metastasis**	

M0	No distant metastasis present

M1	Distant metastasis
